# Daily Alcohol and Cigarette Use Among Sexual Minority Adults: Moderating Role of Daily Stress and Daily Well-Being

**DOI:** 10.1093/geroni/igaa057.3174

**Published:** 2020-12-16

**Authors:** Agus Surachman, Britney Wardecker, Cara Exten, Jes Matsick, David Almeida

**Affiliations:** 1 The Pennsylvania State University, University Park, Pennsylvania, United States; 2 Penn State University, University Park, Pennsylvania, United States; 3 Pennsylvania State University, University Park, Pennsylvania, United States

## Abstract

The goal of this study was two-fold: 1) to investigate whether gay, lesbian, and bisexual (GLB) adults, compared to heterosexual adults, used alcohol and cigarettes daily to a greater extent, and 2) to test the moderating role of daily stress and well-being on the association between GLB status and alcohol and cigarette use. We analyzed data from 3,421 adults (GLB = 98; age range = 20-83 years) who completed an 8-day daily diary protocol as part of the Midlife in the U.S. Study (MIDUS). Compared to heterosexual adults, GLB participants reported greater daily alcohol and cigarette use. However, among GLB individuals, more negative affect was associated with less daily alcohol use and people who reported more stressor days and physical symptoms across the week consumed less alcohol. We will discuss how daily affect, stress, and substance use may function differently among GLB people in middle and older adulthood.

